# Structural and (Pseudo-)Enzymatic Properties of Neuroglobin: Its Possible Role in Neuroprotection

**DOI:** 10.3390/cells10123366

**Published:** 2021-11-30

**Authors:** Giovanna De Simone, Diego Sbardella, Francesco Oddone, Alessandra Pesce, Massimo Coletta, Paolo Ascenzi

**Affiliations:** 1 Dipartimento di Scienze, Università Roma Tre, Viale Marconi 446, 00146 Roma, Italy; giovanna.desimone@uniroma3.it; 2 IRCCS Fondazione Bietti, 00198 Roma, Italy; diego.sbardella@fondazionebietti.it (D.S.); francesco.oddone@fondazionebietti.it (F.O.); 3 Dipartimento di Fisica, Università di Genova, Via Dodecaneso 33, 16100 Genova, Italy; pesce@fisica.unige.it; 4 Dipartmento di Scienze Cliniche e Medicina Traslazionale, Università di Roma “Tor Vergata”, Via Montpellier 1, 00133 Roma, Italy; 5 Accademia Nazionale dei Lincei, Via della Lungara 10, 00165 Roma, Italy; 6 Unità di Neuroendocrinologia, Metabolismo e Neurofarmacologia, IRCSS Fondazione Santa Lucia, 00179 Roma, Italy

**Keywords:** neuroglobin, structure, reactivity, (pseudo-)enzymatic properties, neuroprotection, retina

## Abstract

Neuroglobin (Ngb), the third member of the globin family, was discovered in human and murine brains in 2000. This monomeric globin is structurally similar to myoglobin (Mb) and hemoglobin (Hb) α and β subunits, but it hosts a bis-histidyl six-coordinated heme-Fe atom. Therefore, the heme-based reactivity of Ngb is modulated by the dissociation of the distal HisE7-heme-Fe bond, which reflects in turn the redox state of the cell. The high Ngb levels (~100–200 μM) present in the retinal ganglion cell layer and in the optic nerve facilitate the O_2_ buffer and delivery. In contrast, the very low levels of Ngb (~1 μM) in most tissues and organs support (pseudo-)enzymatic properties including NO/O_2_ metabolism, peroxynitrite and free radical scavenging, nitrite, hydroxylamine, hydrogen sulfide reduction, and the nitration of aromatic compounds. Here, structural and (pseudo-)enzymatic properties of Ngb, which are at the root of tissue and organ protection, are reviewed, envisaging a possible role in the protection from neuronal degeneration of the retina and the optic nerve.

## 1. Introduction

Mammalian tetrameric hemoglobin (Hb) and monomeric myoglobin (Mb) are generally taken as macromolecular models, having been among the first proteins that were characterized from the structural and functional viewpoints. Over the last 30 years, the increase of genomic information and the availability of expression systems opened new horizons in the distribution, folding, and function of all α-helical globins [1,2,3,4,5,6,7,8].

All α-helical globins are expressed in all living organisms, with putative globin genes occurring in many prokaryotes and eukaryotes. In prokaryotes, about 65% of the known bacterial genomes contain one or more globin gene, whereas globin genes have been found in the genome of more than 90% of the eukaryote organisms classified so far [9,10,11,12,13]. In addition to classical 3/3 all α-helical Hb and Mb, several 3/3 and 2/2 α-helical globins have been discovered over the last two decades in vertebrates: (*i*) neuroglobin (Ngb) in neurons and glial cells, (*ii*) cytoglobin (Cygb) in fibroblasts, (*iii*) globin E (GbE) in avian eyes, (*iv*) globin X (GbX) in many metazoans but not in birds and mammals, (*v*) globin Y (GbY) in amphibians and monotreme mammals, and (*vi*) the chimeric heme-protein androglobin (Adgb) [8,10,11,12,13,14,15,16,17,18,19,20]. In humans, Adgb, Cygb, Hb, Mb, and Ngb are expressed in canonical and non-canonical sites fulfilling organ-specific (un)common functions [13].

In 3/3 all α-helical globins, present in unicellular and multicellular organisms, the heme is sandwiched between A-B-E α-helices on one side and F-G-H α-helices on the other side, with the proximal HisF8 residue forming the fifth coordination bond of the heme-Fe atom [2,4,8]. Only in unicellular organisms, all α-helical globins characterized by the 2/2-fold representing a subset of the 3/3 α-helical classical globin fold have been discovered [6,21]. Instead of the six to eight α-helical segments (lettered A to H) observed in the 3/3 structural organization, the 2/2 globin fold is composed of four α-helices (named B, E, G, and H), which are arranged around the heme in a sort of bundle composed of the antiparallel pairs B/E and G/H connected by an extended polypeptide loop. The most striking differences between the 2/2 and the 3/3 globin folds are: (*i*) the drastically shortened or absent A-helix, (*ii*) the alteration of the C-E region, and (*iii*) the presence of a long polypeptide segment, which precedes the short α-helix F where the proximal HisF8 residue is located coordinating the heme-Fe atom [2,4,7,8,21]. Over the last two decades, all β-barrel non-canonical globins (i.e., nitrophorins and nitrobindins) mainly devoted to NO metabolism have been reported [22,23,24,25,26,27,28,29]. Of note, human nitrobindin, corresponding to the *C*-terminal domain of the nuclear protein THAP4, has been hypothesized to act as a NO sensor modulating the transcriptional activity residing at the *N*-terminus [25,26,27,28,29].

Interestingly, cavities and/or tunnels within the protein matrix have been recognized in several members of all α-helical and all β-barrel globins [25,30,31,32,33,34,35,36,37,38]. Although internal cavity/tunnel systems reduce the stability of globins, they play a pivotal role in controlling the diffusion of ligands and possibly substrates to the metal center and the reshaping of the heme pocket [39,40,41].

Beyond the well-known O_2_ transport and storage in Metazoa, globins have been reported to display (pseudo-)enzymatic properties including NO/O_2_ metabolism, gas sensing, peroxidase activity, signaling functions, oxidative stress protection, fatty acid metabolism, anti-apoptotic effects, anti-proliferative and cytoprotective effects in cancer cells, anti-microbial and anti-inflammatory functions, and tissue repair and regeneration [8,10,11,12,13]. Here, the structural and (pseudo-)enzymatic properties of multifaced Ngb along with the possible protective role in the retina and the optic nerve are reviewed [13,42,43,44,45,46,47,48,49].

## 2. The Ngb Structure

**Figure 1:**
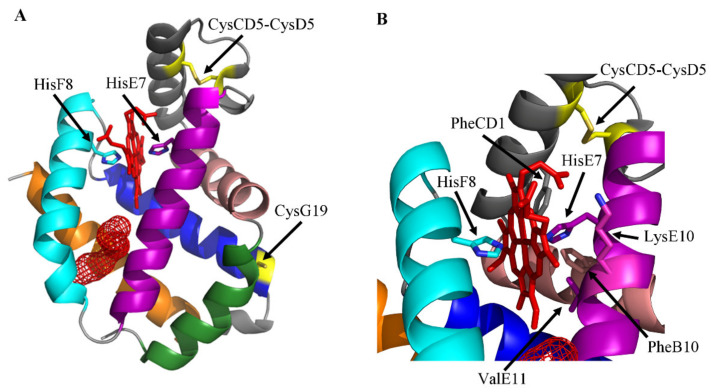
(**A**) Three-dimensional structure of wild-type human Ngb (ID PDB code: 4MPM) [53]. A, B, E, F, G, and H α-helices, forming the classical 3/3 sandwich, are in forest green, salmon, purple, cyan, blue, and orange, respectively. Mesh surfaces of cavity/tunnel systems are highlighted in red. The CysCD5–CysD5 bridge and the CysG19 residue are highlighted in yellow. The proximal and distal amino acid residues HisF8 and HisE7, respectively, axially coordinating the heme-Fe atom through the Nε atom, are shown as balls and sticks. Tunnel/cavity systems have been determined by the interactive software CAVER Web 1.1 [68]. (**B**) Close-up of the heme pocket showing relevant residues cited in the text, in ball and stick representation. Figures drawn by PyMOL package [69].

Only the three-dimensional structures of *Homo sapiens*, *Mus musculus*, and *Symsagittifera roscoffensis* Ngb have been solved. Ngbs from the marine flat worm and mammals display the classical 3/3 globin fold, hosting the heme-group with α-helices A, B, E, F, G, and H, which are organized into a two-layer structure (A-B-E and F-G-H) [32,34,50] superimposable to that of Mb and Hb chains [2,4,8,51]. In ligand-free human and murine Ngb, the heme-Fe atom is six-coordinated by four N atoms belonging to the heme plane; the proximal (or fifth) and the distal (or sixth) ligands of the metal center are HisF8 and HisE7 residues, respectively [32,34]. In human and murine Ngb, the HisE7 imidazole ring is staggered relative to the heme pyrrole N atoms, being oriented toward the methinic bridge CHA and CHC atoms of the porphyrin ring. As a result, hexa-coordination is facilitated, since the α-helix E is pulled toward the heme by about 3.0 Å, relative to sperm whale Mb, which instead displays a five-coordinated heme-Fe atom [2,32,34] (Figure 1A).

The heme distal site of human and murine Ngb is crowded by the apolar residues (PheB10, PheCD1, and ValE11) trapping the porphyrin ring. LysE10 participates as well to the stabilization of macrocycles by electrostatic interactions with heme propionates [32,34]. Both in the crystalline state and in solution, the heme of mutant human and murine ferric Ngb (Ngb(III)) is accommodated in its pocket in two different geometries being rotated by 180° about the α–γ-meso axis [32,34]. The equilibrium constant for the two heme orientations in murine Ngb changes from ~2:1 in ligand-free Ngb(III) to ~1:1 in the Ngb(III)–cyanide complex, suggesting a role in the modulation of heme reactivity [52]. However, the structure of wild-type human Ngb reveals neither the occurrence of the heme isomerization nor the presence of multiple conformations of HisE7 [53]. Of note, the HisE7 and HisF8 imidazole rings of murine Ngb(III) show different orientations in the crystalline state and in solution, reflecting either the high exposure of the heme group to the bulk solvent or the absence of hydrogen-bond acceptors for the His NH protons provided by the heme-protein [54] (Figure 1B).

The overall fold of *Symsagittifera roscoffensis* Ngb1 matches well to that of murine ferrous carbonylated Ngb [35,50]. However, TrpCD3 is hydrogen bonded to one of the heme propionates and may represent a barrier for ligand access to the heme pocket. In addition, a water molecule is hydrogen-bonded to the heme distal HisE7 residue and to the second propionate of the heme group [50]. Unlike most globins, *Symsagittifera roscoffensis* Ngb displays a unique feature in that the α-helix F is bent by the presence of ProEF10, possibly reflecting the heme-protein flexibility [50].

A large cavity/tunnel system, encompassing part of the heme distal and proximal sites, is present in the protein matrix of human and murine Ngb, representing a pivotal determinant for the function and dynamics of these globins. In wild-type human Ngb, the cavity/tunnel system is shielded from the bulk solvent and is lined with hydrophobic residues, including ValE14, IleE15, ValG8, LeuG12, LeuH11, and ValH15. The volume of this cavity is 89 Å^3^ and 225 Å^3^ in molecules A and B present in the asymmetric unit, respectively. The different cavity size observed in A and B monomers of wild-type human Ngb is not a crystallographic packing artifact but reflects the high flexibility of the CD region that is crucial in the modulation of the heme reactivity and in the reshaping of the internal cavities [53]. In addition, a solvent-inaccessible internal cavity connected to the heme distal pocket (~60 Å^3^) occurs in the neighborhood of the CD loop/α-helix D region [53]. In the ligand-free CysCD5Gly/CysD5Ser/CysG19Ser mutant of human Ngb, a protein cavity (~120 Å^3^) connects the heme distal pocket to the EF inter-helical hinge. This cavity is lined with hydrophobic residues provided by the B, E, G, and H α-helices. Although in the crystals the cavity appears to be shielded from the external solvent, the intramolecular contacts indicate that, in a dynamic context, solvent access may be gained through side chain fluctuations [32]. In the ligand-free CysD5Ser/CysG19Ser mutant of murine Ngb, two small cavities (16 Å^3^ and 11 Å^3^), are present on the heme distal side. Moreover, a highly hydrophobic cavity (287 Å^3^), responsible for the unique CO binding mode [34,35], connects the heme distal and proximal sides [34], being also connected with the external solvent through a channel. In fact, upon CO binding, heme slides towards the interior of the protein matrix, decreasing the structural disorder especially in the EF-F-FG region, and inducing peripheral structural changes, which may affect Ngb actions and partner recognition. The repositioning of the heme-Fe group affects the geometry of HisF8, always bound to the heme-Fe(II) atom, and of the PheG5 [35]. The unusual CO-dependent repositioning of the heme group avoids the necessity of a large HisE7 swinging motion to permit ligand entry in and exit from the heme distal pocket [35], which is instead observed in Mb and Hb (i.e., the so-called “E7 gate” mechanism) [55,56,57]. Although the orifice of the channel is narrow, diatomic ligands can nonetheless access the metal center by virtue of the flexibility of the EF corner contributing to the channel external wall [34,35,58,59,60]. Due to the larger internal packing defects and the more pronounced active site fluctuations, the CO transfer barriers are considerably lower in Ngb(II) as compared to Mb(II) [61,62,63,64,65,66] (Figure 1).

In ferrous carbonylated murine Ngb, the CO molecule is bound to the heme-Fe(II) atom in a bent configuration, the Fe–C–O angle ranging between 100 and 156° and the Fe–C and Fe–O distances range between 1.8 to 2.0 Å and 2.5 to 3.0 Å, respectively. Moreover, the O atom of CO is placed at 2.8–3.2 Å from the NE atom of HisE7, possibly being stabilized by hydrogen bonding [35,58,60,67].

In ferrous oxygenated *Symsagittifera roscoffensis* Ngb1, O_2_ is bound to the distal site of the heme-Fe atom in a bent configuration, the Fe–O1–O2 angle being 136°. Moreover, the Fe–O1 and Fe–O2 distances are 1.96 and 3.05 Å, respectively, and the O1 and O2 atoms of O_2_ are located at 3.00 and 3.25 Å, respectively, from the NE atom of HisE7, which in turn stabilizes the heme-Fe-bound O_2_ by hydrogen bonding [50].

Mutagenesis on the structure of human and murine Ngb was relevant to understand at the atomic level the reactivity properties of this monomeric globin and to highlight the potential in vivo role of the redox modulation of the CD5–D5 disulfide bond.

## 3. Ngb (Pseudo-)Enzymatic Properties

Ngb is present at a relatively low concentration (~1 μM) compared to that of Mb (~200–400 μM) in most tissues and organs; this suggests that the role of Ngb in O_2_ supply and/or facilitated diffusion may become significant only in the retinal ganglion cell (RGC) layer and in the optic nerve where the Ngb concentration is up to ~100–200 μM [18,70,71,72,73]. Despite its low concentration, Ngb displays (pseudo-)enzymatic properties including NO/O_2_, peroxynitrite, and free radical scavenging; nitrite, hydroxylamine, and hydrogen sulfide reduction; and the nitration of aromatic compounds [44].

Of note, the heme-based reactivity of human Ngb is allosterically modulated by the redox state of the cell. In fact, the six-to-five-coordination transition (i.e., the inactive-to-active state) of the heme-Fe atom depends on the cleavage or the formation of the CysCD5–CysD5 bond, belonging to the so-called “dynamic loop” (CD loop/α-helix D region), and the (de-)phosphorylation of SerA7, SerA12, SerAB2, SerCD10, SerCD11, and SerDE3 [32,53,74,75,76,77].

## 4. The Cleavage of the HisE7-Heme-Fe Bond Modulates the Ngb Reactivity

**Figure 2:**
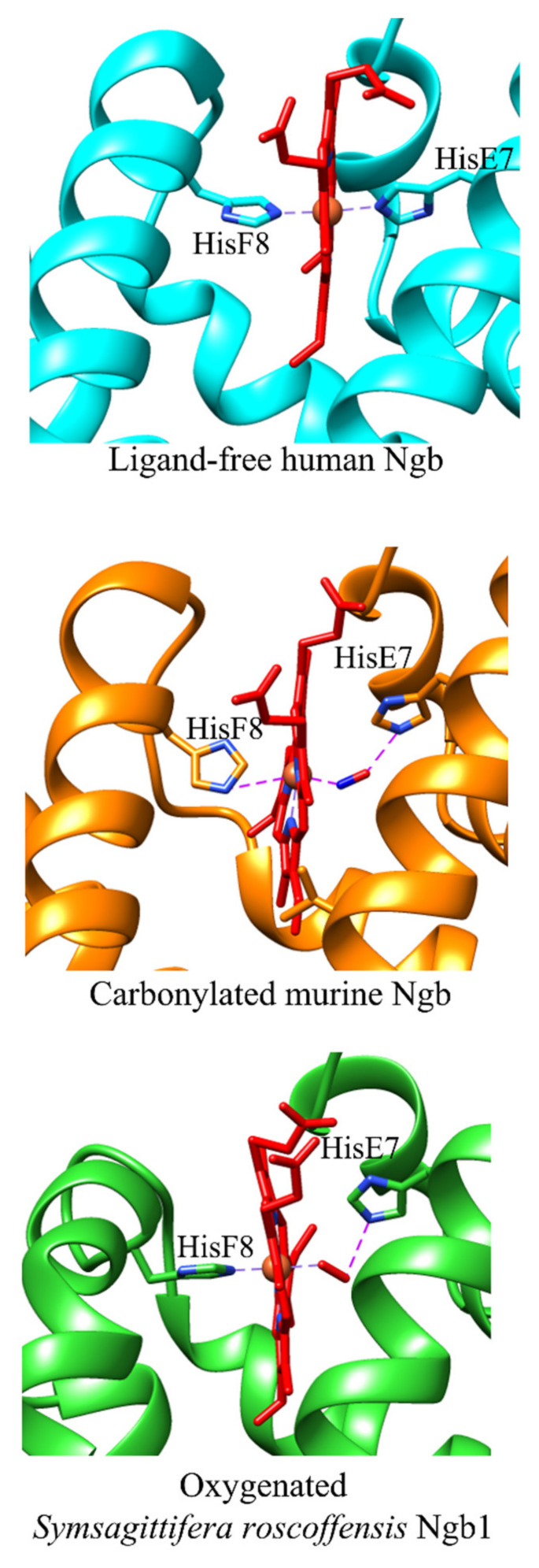
Heme cavities of six-coordinated ligand-free human Ngb (ID PDB code: 1OJ6) [32], carbonylated murine Ngb (ID PDB code: 1Q1F) [35], and oxygenated *Symsagittifera roscoffensis* Ngb1 (ID PDB code: 4B4Y) [50]. All pictures have been drawn by UCSF-Chimera package [84].

The reactivity of six-coordinated human and murine Ngb is modulated through the minimum three-state mechanism encompassing (*i*) the dissociation of the endogenous ligand HisE7 from the HisF8-Fe-HisE7 complex, (*ii*) the formation of the transient five-coordinated HisF8-Fe adduct, and (*iii*) the association of the exogenous ligand (i.e., L) leading to the formation of the HisF8-Fe-L complex [35,74,78,79,80] (Figure 1 and Figure 2). The rate of HisE7 dissociation (i.e., *k*_−1_) limits the heme reactivity only under conditions where the formation of the transient five-coordinated HisF8-Fe adduct is slower than the rate of exogenous ligand binding to the metal center [8,44,81,82,83]. Of note, the five-coordinated HisF8-Fe adduct of *Symsagittifera roscoffensis* Ngb1 may represent the transient reactive adduct of six-coordinated human and murine Ngb (Figure 1 and Figure 2) [50].

**Figure 3:**
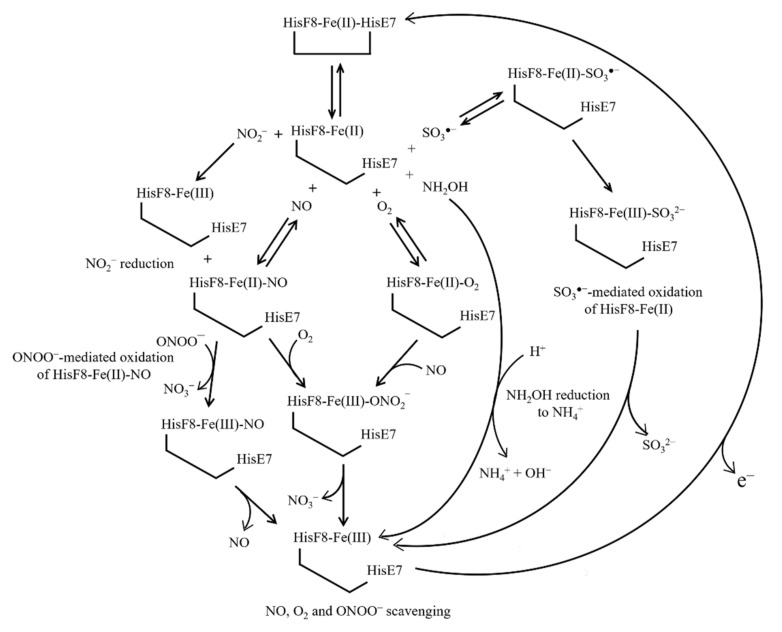
(Pseudo-)enzymatic properties of Ngb(II). The Ngb unreactive hexacoordinated HisF8 Fe(II)-HisE7 species and the reactive pentacoordinated HisF8-Fe(II) conformation are highlighted.

Values of *k*_+1_ for HisE7 binding to human Ngb(II) range from 2.0 × 10^3^ s^−1^ to 9.8 × 10^3^ s^−1^ (pH 7.0 and 20.0–25.0 °C), and the value of *k*_+1_ for HisE7 binding to murine Ngb(II) is 2.0 × 10^3^ s^−1^ (pH 7.0 and 20.0 °C) [85]. Values of *k*_−1_ for HisE7 dissociation from human Ngb(II) range between 1.0 × 10^−1^ s^−1^ and 8.2 × 10^3^ s^−1^ (pH 7.0 and 20.0–25.0 °C), and *k*_−1_ values of murine Ngb(II) range between 1.5 × 10^−1^ s^−1^ and 1.2 s^−1^ (pH 7.0 and 20.0–25.0 °C) [16,44,78,79,80,85,86,87,88,89,90,91]. In the case of Ngb(III) the value of *k*_−1_ for the dissociation of HisE7 is >4.5 × 10^−1^ s^−1^ (pH 7.2 and room temperature) [92]. The different values of *k*_+1_ and *k*_−1_ of human and murine Ngb [32,34,35] reflect, among others, the heme isomerization, the coordination state of the heme-Fe atom, and/or the chemical modifications affecting the heme-Fe reactivity [74,77,93,94]. In particular, the *k*_−1_ value of human Ngb(II) changes from 6.0 × 10^−1^ s^−1^ to 7.0 s^−1^, reflecting the cleavage and the occurrence of the CysCD5–CysD5 bridge, respectively [74]. In contrast, the *k*_−1_ value of murine Ngb(II) (1.0 × 10^−1^ s^−1^; pH 7.0 and 25.0 °C) [76] is unaffected by the redox state of the cell since the CD5 residue is Gly [44]. However, since in many of the following reactions the reduced Ngb(II) species is the most active form, a very important role for the Ngb metabolic function is played by the disulfide redox balance as well as by the kinetics of distal histidine coordination with the heme’s iron (Figure 3) [95].

Interestingly, the heme-Fe reactivity of six-coordinated Ngb is similar to that of five-coordinated globins (e.g., Mb), although it is modulated by the strength of the HisE7-Fe bond (about −3 kcal mol^−1^) rather than by the ligand accessibility to the heme pocket through the E7 gate, possibly reflecting a case of convergent evolution [8,32,34,35,37,38,44,50,53,55,56,57,58,59,67,76,96,97,98,99,100,101,102,103].

## 5. Ngb(II) Oxygenation

The brain is the most energetically demanding organ, but it displays a limited capacity to store energy; therefore, it is highly dependent on O_2_ and glucose supply from the blood stream [8,44,104,105,106,107]. Human and murine Ngb(II) binds reversibly to O_2_ with values of the Hill coefficient *n* close to unity, as expected for monomeric globins [8,44]. Values of *P*_50_ for human Ngb(II) oxygenation range between 0.9 mmHg and 10 mmHg (pH 7.0 and 25.0 °C), thus reflecting the different globin activation states, whereas the *P*_50_ value for murine Ngb(II) oxygenation is 2.2 mmHg (pH 7.0 and 25.0 °C). Since human and murine Ngb(II) oxygenation is limited by the cleavage of the heme-Fe-HisE7 bond, values of the intrinsic dissociation equilibrium constant for O_2_ binding to the transient five-coordinated species are lower than those of the six-coordinated state by about three orders of magnitude [8,14,44,74,78,80,85,86,108,109,110,111,112].

The rate of O_2_ binding to six-coordinated Ngb(II) (i.e., *k*_+2_), as measured by rapid-mixing, is independent of the ligand (e.g., O_2_, CO, and NO) and of the O_2_ concentration, being limited by the cleavage of the heme-Fe-HisE7 bond (i.e., *k*_−1_). Values of *k*_+2_ for human Ngb(II) oxygenation range from 6.0 × 10^−1^ s^−1^ to 7.0 s^−1^ (pH 7.0 and 25.0 °C), reflecting the redox state of the CysCD5/CysD5 residue pair [74]. Since this disulfide bridge is absent in murine Ngb(II), the value of *k*_+2_ for the heme-protein oxygenation (1.5 × 10^−1^ s^−1^; pH 7.0 and 25.0 °C) is independent of the cell redox properties [74,79,80]. The value of the second-order rate constant for the oxygenation of five-coordinated human and murine Ngb(II) (*k*_+3_), as measured after flash and laser photolysis, is ~2.0 × 10^8^ M^−1^ s^−1^ (pH 7.0 and 25.0 °C) and the first-order rate constant for Ngb(II)-O_2_ deoxygenation (i.e., *k*_−3_) is ~6.0 × 10^−1^ s^−1^ (pH 7.0 and 25.0 °C) [74,78,86,113,114] (Figure 3).

## 6. Ngb(II) Nitrosylation

The ubiquitous messenger NO plays pivotal roles in several regulatory functions, including vasodilatation, neurotransmission, and host-defense mechanisms; however, it also displays cytotoxic properties and is involved in a variety of neurodegenerative diseases [115,116]. The reaction of NO with murine Ngb(II), measured by rapid-mixing (i.e., *k*_+4_), is slow (~4.0 × 10^−1^ s^−1^; pH 7.0 and 20.0 °C) and independent of the ligand concentration because it can only occur upon cleavage of the heme-Fe(II)-HisE7 bond limiting the association of the external ligand [79]. Flash photolysis study shows a fast recombination rate (i.e., *k*_+5_ = 1.5 × 10^8^ M^−1^ s^−1^; pH 7.0 and 25.0 °C) and a low dissociation rate (*k*_−5_ = 2.0 × 10^−4^ s^−1^; pH 7.0 and 25.0 °C) for murine Ngb(II) (de)nitrosylation, indicating a high intrinsic affinity for the ligand (*K*_5_ = 1.3 × 10^−12^ M; pH 7.0 and 25.0 °C). However, since NO binding to murine Ngb(II) is affected by competitive inhibition of the HisE7 residue (*K*_1_ ~ 1.0 × 10^−3^; pH 7.0 and 25.0 °C) [74,78], the observed NO affinity decreases by about three orders of magnitude. As expected, NO affinity for ferrous five-coordinated HisE7Val, HisE7Gln, and HisE7Leu mutants corresponds to the intrinsic affinity of the wild-type heme-protein [113] (Figure 3).

## 7. NO and O_2_ Scavenging from Ngb(II)–O_2_ and Ngb(II)–NO

Ngb could be involved in the protection of the brain form damage, either scavenging O_2_ or detoxifying NO [79,92]. Human and murine Ngb(II)–O_2_ and Ngb(II)–NO react with NO and O_2_, respectively, yielding Ngb(III) and NO_3_^−^ as the final products and preventing, among others, protein nitration [79,92,117,118]. The reaction of human and murine Ngb(II)–O_2_ with NO proceeds via the transient intermediate Ngb(III)–ONO_2_^−^, which dissociates mono-exponentially with values of the first-order rate constant (i.e., *k*_−6_) of ~3.6 × 10^2^ s^−1^ and ~3.0 × 10^2^ s^−1^, respectively (pH 7.0 and 20.0 °C) [79,118]. O_2_ detoxification from human Ngb(II)-NO is a biphasic process, with values of the second-order rate constant (*k*_+7_) being 1.6 × 10^1^ M^−1^ s^−1^ and 4.0 × 10^−1^ M^−1^ s^−1^ (pH 7.0 and 25.0 °C) [92]. Since values of the bimolecular rate constant for O_2_ and NO binding to five-coordinated human Ngb(II) are almost identical (*k*_+3_ ~ *k*_+5_ ~ 10^8^ M^−1^ s^−1^), the in vivo formation of Ngb(II)–NO is unlikely, occurring only under conditions where NO levels exceed those of O_2_ [79] (Figure 3).

## 8. NO_2_^−^ Reduction by Ngb(II)

Human Ngb(II) catalyzes the reduction of NO_2_^−^, leading to the formation of Ngb(III), Ngb(II)-NO, and NO. This process is modulated by the redox-sensitive CysCD5 and CysD5 residues regulating the fraction of the highly reactive five-coordinated state of the heme-Fe(II) atom. Therefore, the value of the second-order rate constant (*k*_+8_) for NO_2_^−^ reduction by human Ngb(II) increases from 6.2 × 10^−2^ M^−1^ s^−1^ to 1.2 × 10^−1^ M^−1^ s^−1^ (pH 7.4 and 25.0 °C) upon formation of the CysCD5–CysD5 disulfide bond. This small effect was observed in vitro and may not be significant in vivo.

Investigations based on the PheB10Val, PheB10His, PheB10Trp, PheB10Leu, HisE7Ala, HisE7Gln, HisE7Trp, ValE11Ala, ValE11Phe, and ValE11Ile mutants indicate that the reductase activity of human Ngb(II) depends on the heme accessibility of NO_2_^−^ [119,120]. Moreover, phosphorylation at putative sites SerA7, SerA12, SerAB2, SerCD10, SerCD11, and SerDE3 by intracellular kinases (e.g., ERK and PKA) increases the nitrite reductase activity of human Ngb(II) by stabilizing the five-coordinated heme-Fe(II) atom. Of note, the binding of the scaffold protein 14-3-3 at putative sites ArgA9-ProAB3 and ArgCD7-ProD1 of human Ngb stabilizes the phosphorylated derivative, enhancing the nitrite reductase activity [77]

The value of *k*_+8_ for NO_2_^−^ reduction by murine Ngb(II) is 5.1 M^−1^ s^−1^ (pH 7.4 and 25.0 °C). Ngb(III) is the main product of the reaction, Ngb(II)–NO being lower than 20%. Upon reaction with nitrite, murine Ngb undergoes *S*-nitrosylation, the CysD5 residue representing the primary site. However, the overall intracellular physiological concentration of NO_2_^−^ (<1 × 10^−5^ M) appears too low to convert Ngb to its *S*-nitrosylated derivative acting as a NO depot, but it may play a role in redox signaling pathways [121] (Figure 3).

## 9. NH_2_OH Reduction by Ngb(II)

Hbs reduce nitrite and hydroxylamine, an intermediate of nitrite reductase, to nitric oxide under anaerobic conditions. However, plant and cyanobacterial Hbs catalyze the reduction of hydroxylamine to ammonium at rates 100–2500 times faster than animal globins, including Ngb. These results support the view that plant and cyanobacterial hemoglobins contribute to anaerobic nitrogen metabolism in support of anaerobic respiration and survival during hypoxia [122,123,124]. Under anaerobic conditions, human Ngb(II) reduces NH_2_OH to NH_4_^+^, being converted to Ngb(III). The second-order rate constant for hydroxylamine reduction to ammonium by human Ngb(II) (*k*_+8_) is <2.5 × 10^1^ M^−1^ s^−1^ [122] (Figure 3).

## 10. Ngb(II) Oxidation by ONOO^−^ and SO_3_^●−^

Peroxynitrite induces the oxidation of human Ngb(II)–NO by means of the Ngb(III)–NO transient species that decays to Ngb(III) and NO. The oxidation of Ngb(II)-NO to Ngb(III)–NO depends linearly on the ONOO^−^ concentration, whereas the dissociation of the Ngb(III)-NO complex is a monomolecular process (Figure 3). The value of the second-order rate constant for the ONOO^−^-dependent oxidation of Ngb(III)-NO (*k*_+7_) is 1.3 × 10^5^ M^−1^ s^−1^ and the value of the first-order rate constant of the Ngb(III)–NO dissociation (*k*_−7_) is 1.2 × 10^−1^ s^−1^ (pH 7.2 and 20.0 °C) [92]. Murine Ngb(II) undergoes oxidation by SO_3_^●−^ sulfur trioxide, yielding Ngb(III) and SO_3_^−^ as final products; the bimolecular rate constant (i.e., *k*_+8_) is 2.6 × 10^9^ M^−1^ s^−1^ (pH 7.2 and 37.0 °C). Since SO_3_^●−^ scavenging by Ngb(II) is inhibited by glutathione, this reaction may take place in the retina where Ngb(II) levels are exceeding those of glutathione [125] (Figure 3).

## 11. Reductive Nitrosylation of Ngb(III)

**Figure 4:**
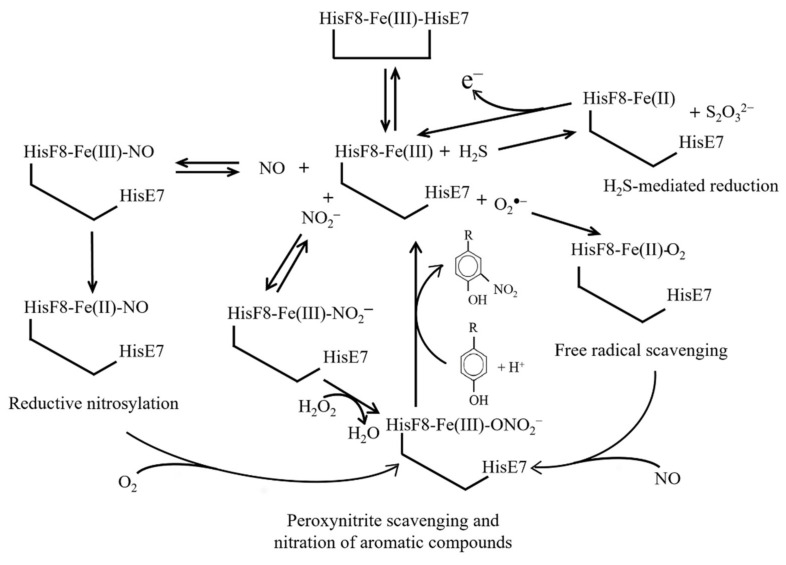
(Pseudo-)enzymatic properties of Ngb(III). The Ngb unreactive hexacoordinated HisF8-Fe(III)-HisE7 species and the reactive pentacoordinated HisF8-Fe(III) conformation are highlighted.

The reaction of human Ngb(III) with an excess of NO leads to Ngb(II)–NO via the formation of the transient Ngb(III)–NO adduct. Values of the dissociation equilibrium constant (*K*_9_) and of the second-order rate constant (*k*_+9_) for NO binding to human Ngb(III) are 7.5 × 10^−5^ M and 2.0 × 10^3^ M^−1^ s^−1^, respectively (pH 7.0 and 20.0 °C) [118]. The value of the first-order rate constant for the dissociation of NO from Fe(III)–NO (i.e., *k*_−9_) is 1.2 × 10^−1^ s^−1^ [92]. The reductive nitrosylation of human Ngb(III) is a biphasic process; values of the overall second-order rate constant of the fast and the slow components (i.e., *k*_+10_) are 2.1 × 10^1^ M^−1^ s^−1^ and 2.9 M^−1^ s^−1^ (pH 7.0 and 25.0 °C), respectively. The transient Fe(III)–NO species converts to Fe(II)–NO with a mono-exponential process, the value of the first-order rate constant (i.e., *k*_+11_) being > 2.4 × 10^−2^ s^−1^ (pH 7.0 and 25.0 °C) [92] (Figure 4).

## 12. H_2_S-Mediated Reduction of Ngb(III)

Hydrogen sulfide H_2_S is a potential signaling molecule produced by cystathionine β-synthase, cystathionine γ-lyase, and 3-mercaptopyruvate sulfurtransferase. H_2_S affects neurotransmission in the brain and relaxes vascular smooth muscle in synergy with NO. Moreover, it has been reported that H_2_S acts as an O_2_ sensor, influences autophagy, displays a cytoprotective and angioprotective effect during the evolution of myocardial infarction, and has anti- inflammatory activity [126]. In addition, the hydrogen polysulfides (H_2_Sn, *n* ≥ 2), produced by 3-mercaptopyruvate sulfurtransferase, are potential signaling molecules able to modulate the activity of ion channels and enzymes as well as the tumor growth by *S*-sulfuration of target proteins [127] (Figure 4).

Under anaerobic conditions, human Ngb(III) catalyzes the oxidation of HS^−^ to S_2_O_3_^2−^ via the formation of the transient Ngb(II)–HS adduct; this reaction exhibits biphasic kinetics. For the fast phase, values of the second-order rate constant for the formation of the Ngb(II)–HS complex (*k*_+12_), of the first-order rate constant for the Ngb(II)–HS adduct dissociation (*k*_−12_), and of the equilibrium dissociation constant for H_2_S binding to Ngb(III) (*K*_12_) are 1.4 × 10^1^ M^−1^ s^−1^, 5.1 × 10^−3^ s^−1^, and 3.7 × 10^−4^ M (pH 7.4 and 25.0 °C), respectively. On the other hand, the rate constant of the slow step for Ngb(II)–HS complexation (*k*_+13_ = 5.6 × 10^−4^ s^−1^; pH 7.4 and 25.0 °C) is independent of the ligand concentration. The Ngb(III) mutant HisE7Ala was more active for H_2_S oxidation (*k*_+14_ = 1.4 × 10^1^ M^−1^ s^−1^, *k*_−14_ = 5.1 × 10^−3^ s^−1^, and *K*_14_ = 3.7 × 10^−4^ M; pH 7.4 and 25.0 °C), yielding the formation of 2–6 catenated sulfur atoms with or without oxygen insertion. This highlights the role of the heme distal residue in modulating the reactivity of the metal center and the different reaction options [127,128,129,130,131] (Figure 4).

## 13. Free Radical Scavenging from Ngb(III)

Human Ngb(III) scavenges a variety of free radicals including O_2_^●−^. The value of the affinity constant (*K*_15_) for O_2_^●−^ binding to Ngb(III) is 7.4 × 10^−6^ M. The CysCD5–CysD5 bond is not necessary to scavenge superoxide, but it plays a pivotal role in stabilizing the heme-Fe(II)–O_2_ complex [132]. Wild-type human Ngb(III) and the PheB10Leu mutant are much more stable in the presence of H_2_O_2_ than the HisE7Leu mutant, which undergoes tyrosyl radical formation. This indicates that (*i*) the PheB10 residue is essentially insensitive to the redox state of Ngb and (*ii*) the five-coordination of the heme-Fe atom, occurring in the HisE7Leu mutant, induces the destabilization of the globin fold [133]. As a whole, the redox changes of the heme-Fe atom and of the CysCD5/CysD5 residue pair could regulate neuroprotective functions of human Ngb, protecting against hypoxic and ischemic stress in the brain [132,134] (Figure 4). Of note, human Ngb(III) does not produce the oxidizing oxoFe(IV)=O species when treated with H_2_O_2_, in contrast to Hb and Mb [92].

## 14. Nitration of Aromatic Compounds by Ngb(III)-NO_2_^−^

Nitrosylated tyrosines play a role in the redox regulation of metabolism; however, their increase occurs in multiple neurodegenerative diseases (e.g., Parkinson’s and Alzheimer’s diseases) [135]. Human Ngb(III)–NO_2_^−^ catalyzes the nitration of phenolic substrates in the presence of H_2_O_2_ via the transient formation of the highly reactive Ngb(III)–ONOO^−^ species (Figure 4). The two human Ngb forms, with and without the internal CysCD5–CysD5 disulfide bridge, reflecting the five- and six-coordination of the heme-Fe(III) atom, respectively, exhibit very different reactivities. In fact, values of the dissociation equilibrium constant (i.e., *K*_16_) and of the second-order rate constant (i.e., *k*_+16_) for the nitration of aromatic compounds by the highly reactive Ngb(III)–ONO_2_^−^ complex are 4.3 × 10^−^^2^ M and 3.3 M^−^^1^ s^−^^1^, respectively, whereas those of the slowly reactive form are 2.2 × 10^−^^1^ M and 6.3 × 10^−^^1^ M^−^^1^ s^−^^1^, respectively [136]. This evidence reinforces the relevance of the cysteine redox equilibrium in Ngb, which plays an important role in addressing its reactivity toward alternative metabolic pathways (Figure 3 and Figure 4).

## 15. Possible Patho-Physiological Roles of Neuroglobin

Up to now, Ngb-based reactions have been investigated separately without attempting to link them to each other. Here, this limitation has been tentatively overcome to support the view that Ngb plays multiple roles in health and disease [44,137,138,139,140].

Ngb was first documented to be almost exclusively expressed in the brain, displaying high concentrations (up to 100–200 μM) in the retina layers, not only in the RGC layer, but also in astrocytes and in Müller cells (i.e., a specialized microglial lineage which spans across the layers, serving protective and nourishing activities). In this last cell type, the Ngb expression is further significantly raised during reactive gliosis in response to eye injury [141].

Ngb actions in vivo depend on its concentration and on an efficient Ngb(III) reductase system, still unknown, restoring Ngb(II) [44,79,80,118,142]. Thus, when present at high concentrations (~100–200 μM) Ngb may facilitate O_2_ buffer and transport in the RGC layer and the optic nerve, whereas at low concentrations (~1 μM) Ngb can display only potential enzymatic activities and cell signaling in most tissues and organs, including resting neurons [43,44,143].

Systems biology investigations and numerical simulations confirm that Ngb should play an important role in O_2_ transport rather than in storage; thus, since the retina is most susceptible to hypoxia in the regions of the photoreceptor inner segment and inner plexiform layers, high concentrations of Ngb have the potential to prevent hypoxia and increase the O_2_ uptake by 30–40% [144]. A mutual regulation of Ngb and hypoxia was envisaged by studies on Ngb expression after the induction of ocular hypertension in Wistar rats. Ngb was quickly transcriptionally upregulated in rat retinal layers in response to the acute retinal ischemia injury induced by the elevation of intraocular pressure, suggesting that the globin might play an important role in the metabolic adaptation to hypoxia [145].

Furthermore, decreased survival of Ngb-deficient primary cortical neurons along with increased viability of the same cells in the presence of Ngb overexpression were originally reported in an experimental model of hypoxia in vitro, where Ngb immunostaining was significantly increased in the surviving infarcted area [146]. In accordance with these findings, the intra-cerebro-ventricular administration of a Ngb antisense, but not sense, oligo-deoxy-nucleotide increased infarct volume, worsening the neurological outcome of stroke [147].

The hypothesis of Ngb having a role in reducing the focal ischemia and infarct size was further pushed forward by the development of transgenic mice overexpressing this globin (Ngb-Tg mice). A reduced infarct size was observed in these animals after transient cerebral arterial ligation followed by reperfusion [148]. Surprisingly, in this transgenic animal, Ngb expression was induced also in non-neuronal cells, such as in heart cells, and this was apparently enough to limit the infarct size area after heart arterial ligation and reperfusion, also in an organ which physiologically does not appreciably express the protein [148]. The reduced infarct size in the same Ngb-Tg animal was further confirmed by other authors [149].

Furthermore, the specific effect of increasing the amount of Ngb in retinal cells has been demonstrated by the injection of exogenous Ngb after a transient hypoxia, which turned out to be effective in decreasing the levels of inflammatory chemokines (IL-6, TNFα, IL-1B, RANTES, MCP-1, and VEGF) and microglia activation [150]. This effect appears to be linked to the regulatory effect of Ngb on the Wnt/β-catenin and NF-*k*B signaling pathways, which is exerted mostly through the induction of the proteasomal degradation of Dishevelled-1, a key hub protein eliciting these two processes [151].

Interestingly, endogenous, and exogenous compounds (e.g., hormones and phytochemicals) as well as injuries (e.g., oxidative stress, hypoxia, epilepsy, and ischemia) increase Ngb levels in the brain, allowing it to carry out functions directed to neuroprotection [152,153,154,155,156]. As a matter of fact, the enhanced expression of Ngb has been observed in RGCs after optical nerve injury either through exposure to intense led light [157] or to an oxidative stress [158].

Clues for interpreting the biological role of Ngb come from zebrafish retina, which, unlike mammals, shows regenerative properties of the optic nerve. In this small fish, Ngb mRNA was found to be expressed in amacrine cells, whereas Ngb protein was also detected in the inner plexiform layer. This discrepancy was attributable to the presence of a membrane-penetrating module of zebrafish Ngb which allowed the protein to cross the plasma membrane. Immuno-histochemical studies after optic nerve injury highlighted that Ngb protein levels were increased in both amacrine cells, in correspondence to the nerve ending processes, and presynaptic regions in the inner plexiform layer, suggesting that Ngb might be released by the former cells and picked up by the ganglion cells, wherein it participates in the regeneration of the axon [159].

Although most of details of the mechanisms through which Ngb exerts its neuroprotective activity are still unclear, co-localization studies in the retina have confirmed a strong interaction of the globin with mitochondria, envisaging a role in assisting energy metabolism and in patrolling apoptosis upon release of mitochondrial factors, such as cytochrome *c*. In this respect, a very important role seems to be played by Ngb in the protection of nerve cells from apoptosis, mainly through its interaction with cytochrome *c* [118,160], which can block the activation of caspase 9 by ferric cytochrome *c* [161,162]. This anti-apoptotic function requires a reduced Ngb(II) which interacts with ferric cytochrome *c* through negatively charged residues (mostly Asp73 and to a reduced extent Glu60, Asp63, and Glu87); this complex quickly reduces cytochrome *c*, impairing its pro-apoptotic role [163].

Some clues strengthening the contribution of Ngb in serving mitochondria functionality come from studies on the Harlequin mouse phenotype, which finds its genetic roots in the depletion of the apoptosis-inducing factor, and recapitulates hallmarks of human neurodegenerative mitochondrial pathologies, such as the age-dependent degeneration of the retina, optic nerve, cerebellum, and cortical regions. Rescue of Ngb expression in the retina, which significantly drops in the brain of the Harlequin mouse upon delivery of an adenovirus associated vector (AAV), improved RGC survival and overall retina neurodegeneration, suggesting that restoring adequate Ngb levels was enough to improve the mitochondrial chain respiration and energy homeostasis [43].

Similarly, the development of Ngb-transgenic (Ngb-Tg) mice has provided a significant technological advancement to point out the effect of Ngb overexpression on mitochondrial dynamics during retinal ischemia in vivo. Remarkably, compared with wild-type animals, Ngb-Tg mice showed a relevant decrease of mitochondrial DNA damage after 7 days of reperfusion following ischemia induction. In accordance with the general neuroprotective activity of Ngb, apoptosis markers, such as the release of the caspase-3 active fragment, were decreased in Ngb-Tg mice and TUNEL assay showed a reduced frequency of apoptotic cells [152].

Further, several studies have suggested that mitochondrial dysfunction may induce retinal degenerative disorders, such as glaucoma, and that Ngb is among the genes transcriptionally downregulated during the glaucoma onset [143]. For this, the role of Ngb has been investigated in a well-established model of retinal neurodegeneration, such as the DBA/2J mice (which develop spontaneous intraocular pressure and RGC degeneration, eventually inducing glaucoma), following the effect of Ngb upregulation in RGCs via a single intravitreal injection of an AAV vector. In these animals, because of decreased enzymatic activities of mitochondrial chain complexes, mitochondrial dysfunction precedes RGC loss by about 5 months, but AAV-Ngb administration led to a general and robust improvement of RGC functionality. Furthermore, immuno-histochemical studies of retina sections indicated the presence of conserved RGC morphology, suggesting neuronal remodeling and synaptic plasticity. Moreover, overexpression of Ngb attenuated ocular hypertension-induced superoxide production and the associated decrease of ATP levels in mice, suggesting that Ngb acts as an endogenous neuroprotectant to reduce oxidative stress and improve mitochondrial function, thereby promoting RGC survival. Thus, Ngb may modulate RGC susceptibility to glaucomatous neural damage [42].

## 16. Conclusions and Perspectives

The neuroprotective role of Ngb is openly debated since differences in transcript and protein Ngb levels have been reported [149,164,165,166,167,168]. Several studies in Ngb-null mice reported that Ngb expression in the central nervous system and in the retina, rather than diffuse, is restricted to specific brain regions and to two out of the ten layers forming the retina [149,169].

The use of Ngb-null mice turned out to be important to highlight some important Ngb functions, since they appear to have a reduced activity of respiratory chain complexes I and III, the degeneration of primary rat RGCs, and the impairment of visual functions [50]. In addition, a 56–60% increase of the infarct volume after focal cerebral ischemia has been observed in Ngb-null mice compared to *wild-type* animals [146]. In turn, the reduction in infarct size induced by ischemia has been obtained by increasing the Ngb levels before the onset of stroke [170]. These data highlight the role of Ngb as a compensatory protein (e.g., stress-sensor and stress-inducible macromolecule) responding to hypoxic/ischemic/oxidative injuries by activating survival/antiapoptotic pathways [171,172].

In the future, a better understanding of the molecular mechanisms at the root of the biological functions of Ngb will bear fundamental and translational significance, especially in the development of therapeutics against stroke and neurological disorders. Ngb-based therapeutic approaches to neurological disorders have started, and the results obtained are promising since no side effects of Ngb overexpression in transgenic mice have been observed [148,173]. However, since Ngb may act as a compensatory protein even in cancer cells, the possibility that systemic Ngb overexpression could enhance cancer cell survival should be taken into account considered; in this respect, the possibility of Ngb gene therapy is being considered realistically [172,174].

## Data Availability

Not applicable.

## Abbreviations

AAV	Adenovirus associated vector
Adgb	androglobin
Cygb	cytoglobin
Hb	hemoglobin
Mb	myoglobin
Ngb(II)	ferrous Ngb
Ngb(III)	ferric Ngb
GbE	globin E
GbX	globin X
GbY	globin Y
Mb	myoglobin
Ngb	neuroglobin
mice Ngb-Tg	transgenic mice overexpressing Ngb
RGC	retinal ganglion cell
